# The Salford Lung Study protocol: a pragmatic, randomised phase III real-world effectiveness trial in chronic obstructive pulmonary disease

**DOI:** 10.1186/s12931-015-0267-6

**Published:** 2015-09-04

**Authors:** Nawar Diar Bakerly, Ashley Woodcock, John P. New, J. Martin Gibson, Wei Wu, David Leather, Jørgen Vestbo

**Affiliations:** Salford Royal NHS Foundation Trust, Salford, UK; Institute of Inflammation and Repair, Manchester Academic Health Science Centre, University of Manchester, Manchester, UK; GlaxoSmithKline, Research Triangle Park, Durham, NC USA; GSK Respiratory Centre of Excellence, GlaxoSmithKline UK Ltd, Uxbridge, UK; Centre for Respiratory Medicine and Allergy, 2nd Floor Education and Research Centre, University Hospital of South Manchester NHS Foundation Trust, Manchester, M23 9LT UK

## Abstract

**Background:**

New treatments need to be evaluated in real-world clinical practice to account for co-morbidities, adherence and polypharmacy.

**Methods:**

Patients with chronic obstructive pulmonary disease (COPD), ≥40 years old, with exacerbation in the previous 3 years are randomised 1:1 to once-daily fluticasone furoate 100 μg/vilanterol 25 μg in a novel dry-powder inhaler versus continuing their existing therapy. The primary endpoint is the mean annual rate of COPD exacerbations; an electronic medical record allows real-time collection and monitoring of endpoint and safety data.

**Conclusions:**

The Salford Lung Study is the world’s first pragmatic randomised controlled trial of a pre-licensed medication in COPD.

**Trial registration:**

Clinicaltrials.gov identifier NCT01551758.

## Introduction

Double-blind randomised controlled trials (RCTs) in chronic obstructive pulmonary disease (COPD) have indicated that inhaled corticosteroids (ICS) combined with a long-acting β_2_-agonist (LABA) are more effective than the individual components in managing stable COPD, reducing exacerbations and improving lung function and health status [1]. However, double-blind RCTs differ from real life in having highly selective eligibility criteria, and enrolling participants who are not representative of patients in clinical practice and have much higher adherence [2]. The once-daily combination of the ICS fluticasone furoate (FF) and the novel LABA vilanterol (VI) (Relvar®) in a patient-friendly dry-powder inhaler (DPI) (Ellipta®) has the potential for improved adherence over the currently available twice-daily ICS/LABA combinations, with improved clinical effectiveness in a real-world setting [3].

The Salford Lung Study (SLS) is the world’s first pragmatic RCT (pRCT) of an investigational medication. SLS will evaluate the effectiveness and safety of the FF/VI combination compared with existing maintenance therapy in a large, real-world population of patients with COPD in conditions of normal care. The study is being conducted in and around Salford, UK. Salford has a high prevalence of COPD in a community served by a single hospital and an established electronic medical record (EMR), connecting both primary and secondary care. Pharmacies also collaborate to allow patients to collect study medication from their usual community pharmacy.

## Methods

### Study design

SLS is a 12-month, open-label, phase III pRCT, evaluating the effectiveness and safety of FF/VI (Relvar®; 100 μg/25 μg once daily, delivered by a novel DPI, Ellipta®) in patients with COPD (clinicaltrials.gov identifier NCT01551758). The study is conducted in accordance with the International Conference on Harmonisation, Good Clinical Practice (GCP), all applicable data protection requirements and the ethical principles outlined in the Declaration of Helsinki 2008. The study was approved by the Ethics committee, National Research Ethics Service Committee North West, Greater Manchester South.

### Patients

All patients with COPD at 66 primary care sites (at the time of manuscript preparation) in and around Salford and South Manchester are identified by their general practitioner (GP) from practice databases and invited to participate in the study.

Eligibility criteria include:

- aged ≥40 years
- documented GP diagnosis of COPD
- regular maintenance inhaler therapy (ICS alone or in combination with long-acting bronchodilator, one or more long-acting bronchodilators, or triple therapy [ICS/LABA plus long-acting muscarinic antagonist])
- at least one COPD exacerbation in the last 3 years.

Minimal exclusion criteria:

- an exacerbation within the previous 2 weeks
- chronic oral corticosteroid use.

**Fig. 1:**
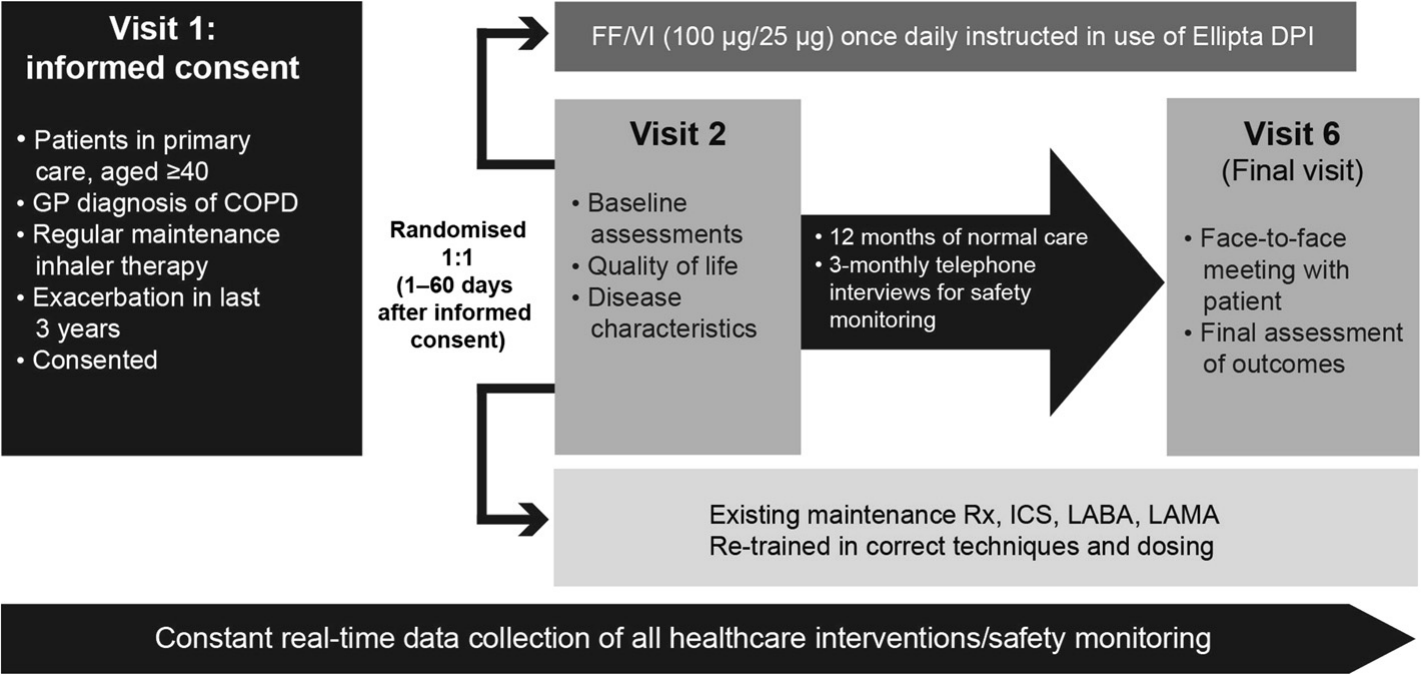
Study design. COPD = chronic obstructive pulmonary disease; DPI = dry-powder inhaler; FF = fluticasone furoate; GP = general practitioner; ICS = inhaled corticosteroid; LABA = long-acting β_2_-agonist; LAMA = long-acting muscarinic antagonist; Rx = treatment; VI = vilanterol

At visit 1, patients are offered study participation through written informed consent (Fig. 1). At visit 2 (1–60 days after visit 1), patients are randomised (1:1) to receive either FF/VI or to continue their usual maintenance therapy. Patients randomised to FF/VI are instructed in the use of the Ellipta DPI. Patients randomised to their usual maintenance therapy are re-trained in the correct techniques and dosing. Baseline assessments are performed at visit 2, including quality of life and disease characteristics (e.g., disease duration, COPD maintenance therapy, smoking status, lung function, medical history). If at months 3, 6 and 9 the patient has had no contact with their GP practice within the previous 8 weeks, the patient is contacted by telephone to assess any serious adverse events (SAEs) or non-serious adverse drug reactions (ADRs) (visits 3, 4 and 5). There is no additional intervention at these assessments. At 12 months, the final visit (6) is a face-to-face meeting with the patient at which the final assessment of outcomes is conducted.

### Participating sites

#### Primary care

To preserve the real-world nature of the study, the patient experience is as close to routine care as possible. The study’s principal investigators are the GPs. They are ideally placed to facilitate recruitment, identify and report SAEs or serious ADRs and report study endpoints. GPs may make treatment adjustments according to their clinical opinion. Repeat prescriptions of study medication are issued by GPs as usual, and collected by patients from their usual pharmacy. As very few participating GPs had experience of clinical trial participation, all GPs have received training and support in GCP, patient recruitment, study protocol, coding of healthcare issues and general research procedures.

#### Pharmacy

Every pharmacy in Salford and others in South Manchester agreed to participate in the study. As with GPs, very few pharmacists had experience of clinical trial participation. All staff (>500) at participating pharmacies have received training in GCP and safety reporting and standard operating procedures were established. Initially, pharmacies faxed copies of study treatment prescriptions to the study coordination centre, but these are now collected electronically. Prescription collection data are used to assess treatment adherence.

#### Hospital

The majority of admissions are to the local hospitals: Salford Royal Hospital and University Hospital of South Manchester. Admissions are identified electronically and assessed by a separate secondary care team within 48 h.

### Data monitoring

All hospital admissions, outpatient and emergency department visits are identified from the EMR database (whenever and wherever they occur). From primary care, all healthcare contacts, out-of-hours activity and prescriptions of antibiotics or oral steroids can be identified. These events are reviewed by the study research team and classified as COPD or non-COPD related. Furthermore, the EMRs capture suspected unexpected serious adverse reactions (e.g., reduced kidney function or elevated liver function tests) and, for the purposes of SLS, include data from external sources to identify, e.g., deaths or National Health Service (NHS) hospital admissions outside Salford. NorthWest EHealth (www.nweh.org.uk) manages the EMRs, enabling data on study endpoints and patient safety to be collected continuously and remotely in near-real time, without the need for face-to-face patient contact.

### Endpoints

#### Effectiveness

The primary endpoint is the mean annual rate of moderate or severe exacerbations. Secondary endpoints include time to first exacerbation and healthcare utilisation. Other endpoints include hospitalisations, use of rescue medication, the COPD Assessment Test (CAT) [4] and EuroQoL-5 dimensions (EQ-5D) questionnaire, listed and defined in Table 1. EMR data for effectiveness endpoints are independently verified by the research team (GP, research nurse, research doctor).

**Table 1:** Study endpoints

Endpoint	Definitions
Primary endpoint	
Mean annual rate of moderate or severe exacerbations	• Moderate exacerbation: patient receiving an exacerbation-related prescription of oral corticosteroids and/or antibiotic (with or without NHS contact) not requiring hospitalisation
	• Severe exacerbation: an exacerbation-related hospitalisation
Secondary endpoints	
• COPD-related secondary care contacts	• Respiratory-related contacts: contact where the most prominent signs and symptoms with which the patient presents are as a direct result of the patient’s COPD
• COPD-related primary care contacts	
	• All contacts: any interaction between the patient and a doctor or nurse working as part of the NHS (including telephone calls), not including protocol-defined study-related visits
• All secondary care contacts	
• All primary care contacts	
• Time to discontinuation of initial therapy	
• Time to addition of a further COPD controller medication	
• Time to first moderate/severe exacerbation	
• Time to first severe exacerbation (i.e., hospitalisation)	
Other endpoints	
• Number of hospitalisations	• Adherence is assessed based on analysis of medications (prescribed, dispensed and collected) and use of the MARS-A at visit 2 and visit 6/early withdrawal visit
• Number of days in hospital	
• Total number of respiratory-related home visits (including out-of-hours calls) and telephone consultations	
• CAT: disease management, quality of life	
• EQ-5D	
• Adherence to study medication	
• Number of salbutamol inhalers collected by the patients from study-enrolled community pharmacies over the 12-month treatment period	

*CAT* COPD Assessment Test, *COPD* chronic obstructive pulmonary disease, *EQ-5D* EuroQol Questionnaire, *MARS-A* Medication Adherence Report Scale for Asthma, *NHS* National Health Service

#### Safety

Safety endpoints include death, pneumonia, frequency and type of SAEs, and ADRs. Investigators and site staff are responsible for detecting, documenting and reporting SAEs and ADRs on electronic case report forms (eCRFs), which are continuously monitored by a dedicated clinical safety team.

### Withdrawals

Patients with COPD exacerbation during the treatment period may remain in the study and continue to take study medication at the discretion of their GP. Severe COPD exacerbations are reported as SAEs. Patients with worsening COPD status while on study treatment may have their medication adjusted at the GP’s discretion or receive other permitted COPD therapies. If, in the investigator’s opinion, the frequency or severity of exacerbations prevents ongoing participation, the patient can be withdrawn. The reason for withdrawal is recorded in the eCRF and patients are followed up for 12 months following randomisation, with their consent.

### Statistical analysis and rationale

The primary efficacy analysis population is intent-to-treat, defined as all randomised patients who have received at least one prescription of study medication.

Sample size calculations are based on the primary endpoint (mean annual rate of moderate and severe exacerbations) and primary efficacy analysis population. A total of 2238 patients (1119 patients per treatment group) are needed. The study has 80 % power to detect a relative reduction of 12 % in the mean annual moderate or severe exacerbation rate, assuming a mean exacerbation rate of 2.3 for the control group [5]. Calculations are based on a negative binomial regression with a dispersion rate of 0.7 and use a two-sided 5 % significance level.

To account for variation in treatment response between patient subgroups, randomisation is stratified by baseline maintenance therapy and by history of COPD exacerbation in the previous 12 months (yes/no) to ensure treatment balance for the primary efficacy analysis population. Analyses based on subgroups are also planned; subgroups will be defined on baseline medication, lung function, comorbidities and other factors.

## Discussion

SLS is a unique pRCT and, to our knowledge, the first prospective real-world comparative effectiveness study of an investigational medicine, which commenced in March 2012, prior to UK regulatory approval (launch date January 2014). The pragmatic inclusion criteria in SLS represent the broad definition of a patient eligible for COPD maintenance therapy in the real world, irrespective of co-morbidities. Study accessibility is maximised by employing minimal exclusion criteria and requirements for additional GP visits. Medicine prescription and supply is achieved as usual, through the patient’s own GP and pharmacy.

Real-world outcomes can be assessed by observational studies that provide high external validity but in contrast have low internal validity [6]. With the limitations in observational studies and those in double-blind RCTs [2] such studies alone may not fully reflect the true impact and value of treatments for COPD. As such, well-designed pRCTs may offer complementary data to these standard types of studies, representing true real-world effectiveness.

Performing a study of a pre-licence drug in a real-world setting has posed many new challenges in study design, operational planning and study support. Patient safety is a priority in studying a pre-licence medicine. Patient safety in SLS is monitored in almost real-time by a combination of remote surveillance of EMRs and clinical monitoring. This sets a new standard, in which safety signals can be seen more quickly than in conventional RCTs. The major challenge has been managing and assessing the relevance and importance of safety signals within the huge volume of healthcare data being generated.

The SLS has limitations. Patients are not blinded and although patients are far less selected than in a usual efficacy trial, some selection bias cannot be precluded. Also, the fact that patients are recruited on the basis of a diagnosis from an electronic medical record and not from a specialist clinic could raise concerns. However, our approach mirrors the real world and a recent study found that registered data had satisfactory validity [7].

## Conclusions

SLS is an innovative project with the aim of evaluating the safety and effectiveness of an investigational medicine in a real-world setting. Data from SLS will allow a better understanding of the risk/benefit profile of the FF/VI combination in the wider COPD community. The study will likely be a role model for future evaluation of effectiveness of new therapies.

## Abbreviations

ADR: Adverse drug reaction
CAT: COPD Assessment Test
COPD: Chronic obstructive pulmonary disease
DPI: Dry-powder inhaler
eCRF: Electronic case report form
EMR: Electronic medical record
EQ-5D: EuroQoL-5 dimensions (EQ-5D) questionnaire
FF: Fluticasone furoate
GCP: Good Clinical Practice
GP: General practitioner
ICS: Inhaled corticosteroid
LABA: Long-acting β_2_-agonist
LAMA: Long-acting muscarinic antagonist
MARS-A: Medication Adherence Report Scale for Asthma
NHS: National Health Service
pRCT: Pragmatic randomised controlled trial
RCT: Randomised controlled trial
Rx: Treatment
SAE: Serious adverse event
SLS: Salford Lung Study
VI: Vilanterol
